# Preparation of Molded Fiber Products from Hydroxylated Lignin Compounded with Lewis Acid-Modified Fibers Its Analysis

**DOI:** 10.3390/polym13091349

**Published:** 2021-04-21

**Authors:** Tianhao Liu, Ying Wang, Jin Zhou, Mengyang Li, Jinquan Yue

**Affiliations:** Key Laboratory of Bio-based Material Science & Technology, Northeast Forestry University, Ministry of Education, Harbin 150040, China; lth15145838960@163.com (T.L.); wangynefu@163.com (Y.W.); zj15927490792@163.com (J.Z.); 15511313386@163.com (M.L.)

**Keywords:** enzymatic hydrolysis lignin, hydroxylation, hemicellulose, molded fiber products, biocomposites

## Abstract

In this study, molded fiber products (MFPs) were prepared from lignin compounded with Lewis acid-modified fibers using enzymatic hydrolysis lignin (EHL) as a bio-phenol. The fibers were modified and compounded entirely through hot-pressing. To improve the reactivity of enzymatic lignin, hydroxylated enzymatic hydrolysis lignin (HEHL) was prepared by hydroxylation modification of purified EHL with hydrogen peroxide (H_2_O_2_) and ferrous hydroxide (Fe(OH)_3_). HEHL was mixed uniformly with Lewis acid-modified fibers on a pressure machine and modified during the molding process. The purpose of Lewis acid degradation of hemicellulose-converted furfural with HEHL was to generate a resin structure to improve the mechanical properties of a MFPs. The microstructure of the MFP was shown to be generated by resin structure, and it was demonstrated that HEHL was compounded on Lewis acid-modified fibers during the molding process. The thermal stability of the MFP with composite HEHL did not change significantly owing to the addition of lignin and had higher tensile strength (46.28 MPa) and flexural strength (65.26 MPa) compared to uncompounded and modified MFP. The results of this study are expected to promote the application of high lignin content fibers in molded fibers.

## 1. Introduction

In recent years, the depletion of fossil resources and the need for sustainable development have led to a great interest in biomass as a renewable energy source. The use of biomass materials (wood, straw, etc.) based on sustainable development principles has significantly improved [1,2]. Biomass composites and their preparation processes have shown high potential owing to their wide range of advantageous properties; among common biomass materials, wood is widely used as a representative renewable biological resource [3]. The raw material of molded fiber products (MFPs) is generally chemical pulp (wood pulp, reed pulp, etc.), which is processed using a unique process and special additives in the mold compression molding of a class of three-dimensional fiber products [4,5]. MFPS have been developed rapidly as packaging materials alternative to plastic and solid wood owning to their high three-dimensional molding capability, good mechanical properties and great modification potential [6,7,8].

The mechanical strength of MFPs is generally thought to result from hydrogen bonding between fibers [9], condensation reactions of lignin in the fiber during the molding process [10], condensation of lignin, and partial degradation of hemicellulose [11]. Therefore, the polymers in the raw materials of MFPs, i.e., cellulose, hemicellulose and lignin, are of particular importance. Cellulose, as the main component of MFPs, should ensure that the fiber properties are not changed significantly during preparation. Therefore, drastic preparation conditions should not be used [12]. As a natural polymeric material with phenylpro-pane as structural monomer, lignin is considered the most promising alternative to phenol-targeted adhesives owing to the presence of hydroxyl groups [13]. The complex structure of lignin as well as the presence of spatial site resistance leads to low reactivity. Increasing the lignin hydroxyl content by chemical means while decreasing the molecular weight facilitates the condensation of lignin in the molding process [14]. Hemicellulose, which is a low molecular weight polysaccharide with a loose structure, is easily hydrolyzed into monomeric compounds (furfural, hydroxymethylfurfural, etc.) during the molding process [15]. Generated furfural-like substances have been reported to replace formaldehyde in the preparation of phenolic resins for composites and to react with phenols in acidic environments to produce biomass-based phenolic resins [16]. Furthermore, addition of lignin has been shown to improve the mechanical properties of MFPs.

In this study, we investigated the effect of a high content of lignin as a filler to be compounded with fibers on the mechanical strength of MFPs. EHL, which is suitable as the MFP filler owing to its high yield and low price, was extracted from corn cob using cellulase degradation. According to the literature, EHL is generally used to prepare lignin-based resins [17,18] and polyurethane foams [19,20], without being studied as a filler compounded into MFPs. Therefore, we focused on the modification and compounding of fibers during the molding process.

## 2. Materials and Methods

### 2.1. Experimental Materials

Enzymatic hydrolysis lignin (EHL) was purchased from Shandong Longlive Bio-technology Co., Ltd. (Shandong, China). Poplar wood chips were taken from the experimental forestry field of Northeast Forestry University. 1,4-Dioxane (AR, 99.7%), ethyl ether (AR, 99.7%), 30% hydrogen peroxide (AR, ≥30%), acetic anhydride (AR, 99.7%), sodium hydroxide (AR, 99.7%), phthalic anhydride (AR, 99.7%), pyridine (AR, 99.7%), phenol (AR, 99.7%), and sodium carbonate (AR, 99.7%) were purchased from Tianjin Fuyu Fine Chemical Co., Ltd. (Tianjin, China). Ferrous hydroxide (AR, 99.8%) and cationic polyacrylamide (CPAM, AR, 99.7%) were purchased from Shanghai Macklin Biochemical Co., Ltd. (Shanghai, China). Folin-Ciocalteu (FC) phenol reagent (1 mol/L) was purchased from Shanghai Lanji Technology Development Co., Ltd. (Shanghai, China). Tetrahydrofuran (THF, HPLC, 99.9%) was purchased from Tianjin Starmark Science and Technology Development Co., Ltd. (Tianjin, China). Ferric chloride (AR, 99.7%) and glacial acetic acid (AR, 99.7%) were purchased from Tianjin Hengxing Chemical Reagent Co., Ltd. (Tianjin, China).

### 2.2. EHL Refining

Raw EHL was sieved using a standard sieve, and particles below 60 mesh were collected and dried in an oven at 80 °C for 12 h. EHL was purified using 1,4-dioxane [21]. EHL (10 g) was dissolved in dioxane/water solution (9/1, *v*/*v*, 100 mL). Once the lignin dissolved completely, the solution was centrifuged, and the filtrate was collected. Then, the filtrate was dropped into ethyl ether (300 mL) and settled in a dark environment for 12 h. The precipitate was collected after washing with deionized water; it was dried at 50 °C under vacuum, grinded and set as reserve.

### 2.3. EHL Hydroxylation Modification

We dissolved 20 g of EHL in 200 mL of sodium hydroxide solution (1%, *w*/*w*) and transferred the solution to a three-necked flask. Then, Fe(OH)_3_ (0.02 g) and 30% H_2_O_2_ (24 g) solution were added under stirring conditions. The reaction was carried out in a water bath (50 °C) after installing a reflux condenser. As reactions completed, the Fe(OH)_3_ residue was removed by centrifugation. The pH of the filtrate was adjusted to 1 with hydro-chloric acid solution (12%, *w*/*w*) to precipitate. The precipitated fraction was washed with deionized water and then freeze-dried and grinded to hydroxylated EHL (HEHL).

### 2.4. Preparation of MFP

Poplar wood chips were impregnated with sodium hydroxide solution (8%, *w*/*v*) for 2 h at 100 °C and then separated into fibers by a split corotating twin-screw extruder to obtain a chemithermomechanical pulp (CTMP) [22].

The base weight of MFP is 1000 g/m^2^. The CTMP fibers (10.5 g) were placed in a mixed solution (300 mL) of acetic acid (3%, *v*/*v*) and FeCl_3_ (0.03 mol/L) for 10 min by maceration. Then, 1 g of CPAM (1%, *w*/*w*) solution was added and dispersed using a fiber sparger. Once the dispersion became uniform, EHL (1.575 g) was added for further dispersion. The mixture was transferred to a mold by Vacuum dehydration and pre-pressure to adjust the moisture content of the sample to 70%. Hot-pressing was carried out at 180 °C and 4 MPa for 20 min to obtain HELS. Table 1 lists the names of the control group samples prepared in this study.

The MFPs listed in Table 1 were pre-pressed using a PL8-B molding equipment (Xianyang Taist Test Equipment Co., Ltd. (Xianyang, China)) and hot-pressed using a ZG-20T pressure machine (Dongguan Zhengyong Electronic Mechanical Ltd. (Dongguan, China)).

### 2.5. Characterization

The micromorphology of the samples was analyzed using scanning electron microscopy (SEM, COXEM EM-30, COXEM Co., Ltd., Daejeon, Korea). Functional group changes were analyzed using Fourier transform infrared (FTIR) spectroscopy (VERTEX-80, Bruker, Billerica, MA, USA). The content of each element of the EHL was analyzed using an Elemantar Vario EL cube (Elementar Analysensysteme GmbH, Langenselbold, Germany) organic elemental analyzer. EHL was acetylated according to the method of Pereira Araujo et al. [23]. The molecular weight of EHL was analyzed using ambient gel permeation chromatograph (GPC, Agilent PL-GPC50 am, Agilent Technologies, Inc., Santa Clara, CA, USA) with polystyrene (PS) specimens with molecular weights of 6,570,000, 3,152,000, 920,000, 468,300, 9820, 4910, 1300, 580 and 162 g/mol. A lignin/THF solution (1 mg/mL) was prepared and insoluble material was removed before performing the GPC assay using a 0.22 μm filter membrane. The flow rate was 1 mL/min and the injection volume was 25 μL. A thermogravimetric analysis (TGA, TGA5500 thermogravimetric analyzer, TA INSTRUMENTS, New Castle, DE, USA) was performed to examine the thermogravimetric curves of EHL and MFPs before and after modification with a sampling mass of 5.5 mg. The test temperature was increased from 30 to 795 °C at a heating rate of 10 °C/min under nitrogen atmosphere (10 mL/min).

The 2D-NMR analysis was made at 25 °C in a Bruker Avance 600 MHz spectrometer (Bruker, Karlsruhe, Germany). Approximately 30 mg of lignin samples were dissolved in 0.50 mL of DMSO-*d*_6_. HSQC experiments used Bruker’s “hsqcetgp” pulse program with spectral widths of 3400 and 18,700 Hz for the ^1^H and ^13^C dimensions. The number of collected complex points was 1024 for the ^1^H dimension with a recycle delay of 1.5 s. The number of transients was 257, and 256 time increments were recorded in the 13C dimension. Processing used typical matched sine square 90° apodization in ^1^H and ^13^C. HSQC spectra were processed with MestReNova, v14.0.0-23239 software (Mestrelab Research, Spain). Prior to Fourier transformation, the data matrices were zero-filling up to 2048 points in the ^1^H and ^13^C dimensions. For correct peak integration, the spectra were previously baseline-corrected with the default option. The central solvent peak was used as an internal reference (δ_C_ 40.03; δ_H_ 2.48).

### 2.6. Determination of Hydroxyl Content

The alcohol hydroxyl content was measured according to ISO14900 (2017). We dissolved 100 mg of EHL in 25 mL of phthalic anhydride acylation reagent. The solution was transferred to a conical flask with a condensing reflux device, which was then placed in a 115 °C oil bath for 0.5 h. We washed the condensing unit with pyridine at the end of the reaction and transferred the solution to a 1000 mL conical flask. Then, the solution was diluted with 500 mL of distilled water; 0.5 mL of phenolphthalein indicator was added and then titrated using sodium hydroxide solution (1.0 mol/L) under magnetic stirring conditions. Moreover, blank experiments were performed as described above.

The phenolic hydroxyl content assay was performed using the FC method [24]. Using a pipette, we added 10 mL of phenol solution (2.14 mmol/L) into 100 mL volumetric flask and fixed the volume. We transferred 0, 1, 2, 4, 8, 10, and 14 mL of the above solution to a 50 mL volumetric flask (the concentrations were 0, 4.278, 8.556, 17.112, 34.224, 42.780, and 59.892 μmol/L, respectively). Then, we added FC reagent (3 mL) and 30, 29, 28, 26, 20, and 16 mL of distilled water, followed by full shaking for 30 s. Further, we added 10 mL of Na_2_CO_3_ (20%, *w*/*v*) solution; then, we mixed the solution uniformly and fixed it with distilled water. The above mixture was reacted with magnetic stirring at room temperature for 2 h to obtain a series of reaction products. The final absorbance of the product was measured at 760 nm and the concentration-absorbance standard curve was plotted.

Next, we accurately weighed 100 mg of dried EHL specimen and dissolved it in 100 mL NaOH (1 g/L, *w*/*v*). We transferred 500 μL of the solution to a 50 mL volumetric flask. Then, we added FC reagent (3 mL) and distilled water (30 mL). After shaking for 5–8 min, we added 10 mL of Na_2_CO_3_ (20%, *w*/*v*) and fixed the solution with 50 mL of distilled water. The mixture was stirred for 2 h before the measurements. The absorbance of a blank sample with a phenol concentration of 0 was used to determine at 760 nm as a reference. The measured absorbance was plotted against the concentration-absorbance standard curve in order to calculate the phenolic hydroxyl content.

### 2.7. Mechanical Properties of MFP

The density of the MFPs was determined according to ISO 534 (2011). Strips of 100 mm × 15 mm were cut for tensile testing, and strips of 100 mm × 25 mm were cut for bending testing. The mechanical strength was tested with a CMT5504 universal strength testing machine (Shenzhen SANS Testing Machine Co., Ltd. (Shenzhen, China)) according to ISO 527-3 (2018) and ISO 178 (2019) standards. The average of five measurements for each group of samples was reported as the result.

## 3. Result and Discussion

### 3.1. Characterization of Lignin

#### 3.1.1. Micromorphology of EHL

**Figure 1:**
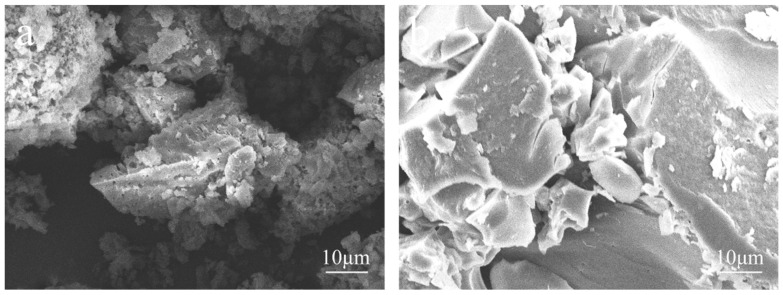
SEM images of (**a**) EHL and (**b**) HEHL at ×2000 magnification.

Figure 1 shows SEM images of the EHL taken to determine the changes in the micromorphology of EHL during the hydroxylation modification process. From the figure, it can be seen that the surface of purified EHL is rough and has many pores, while the surface of HEHL is relatively smooth. It is presumed that the molecular weight of the modified EHL decreases and the content of hydroxyl groups increases, which causes the micromorphology changes due to the degradation of the surface molecules of EHL by H_2_O_2_.

#### 3.1.2. GPC of EHL and HEHL

**Figure 2:**
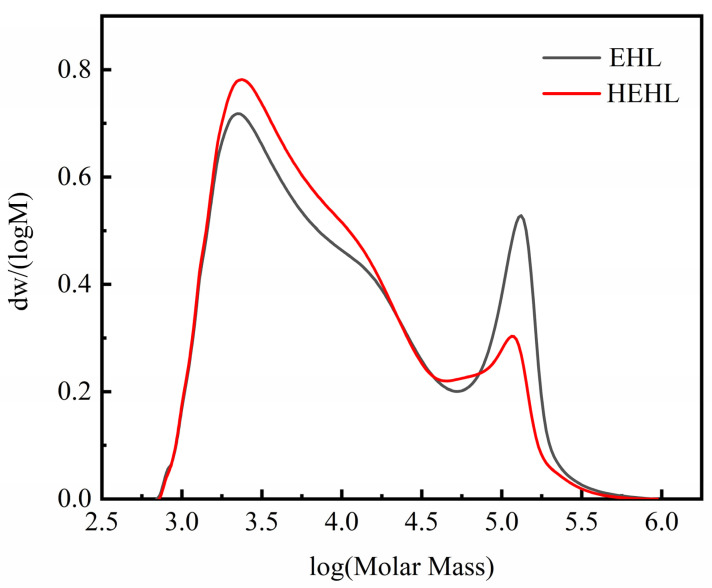
Molecular weight distribution curves of EHL and HEHL samples.

The GPC curves of the soluble components of EHL and HEHL are shown in Figure 2 and Table 2. After hydroxylation modification, M_w_ of EHL decreased from 16,900 to 12,600 and its M_n_ decreased from 2900 to 2800. The decrease in M_w_ indicates the conversion to relatively small molecules during the modification process. The breakage of the ether bond was accompanied by the generation of a large number of hydroxyl groups, resulting in the decrease in the relative molecular mass of EHL. The distribution index (M_w_/M_n_), which is an indicator of polydispersity, of the EHL and HEHL samples measured by GPC, was 5.83 and 4.50, respectively. The decrease in the distribution width index indicates that the relative molecular mass of HEHL is more uniformly distributed. In Figure 2, the area changes of the molecular weight distribution curves of EHL and HEHL indicate that the molecular weight distribution of HEHL becomes more uniform. The uniformity of the relative molecular mass distribution of EHL is beneficial for its application in subsequent experiments.

#### 3.1.3. Hydroxyl Content of EHL

**Figure 3:**
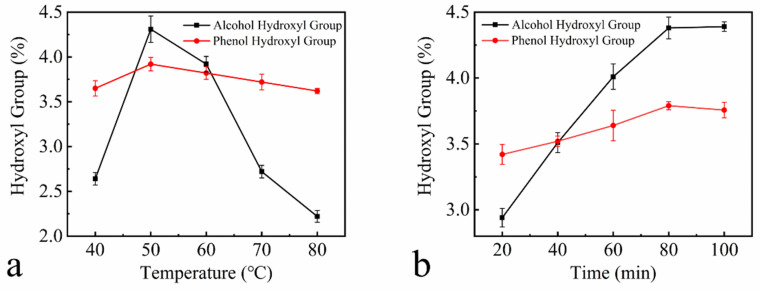

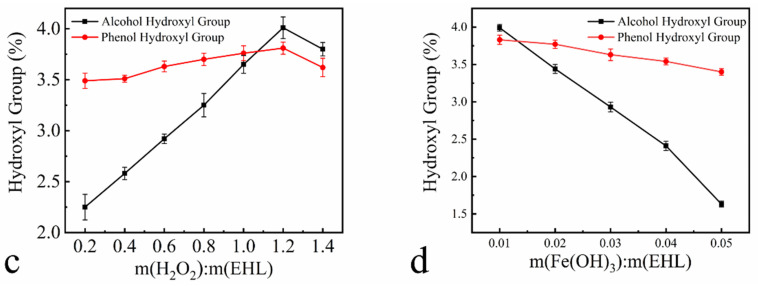
Variation of hydroxyl group content with the following factors: (**a**) temperature at m(H_2_O_2_): m(EHL) = 1:1, m(Fe(OH)_3_): m(EHL) = 0.01:1, and 60 min reaction time; (**b**) time at m(H_2_O_2_): m(EHL) = 1:1; m(Fe(OH)_3_): m(EHL) = 0.01:1, and 50 °C reaction temperature; (**c**) H_2_O_2_ content at m(Fe(OH)_3_): m(EHL) = 0.01:1; 60 °C reaction temperature, and 60 min reaction time; (**d**) Fe(OH)_3_ content at m(H_2_O_2_): m(EHL) = 1:1, 60 °C reaction temperature, 60 min reaction time. All data in Figure 3 are based on three parallel experiments.

Figure 3 shows plots of the hydroxyl content of HEHL as a function of the reaction temperature, time and material/liquid ratio. The experimental parameters for the hydroxylation modification of EHL are 50 °C, 80 min, 1.2 mass ratio of H_2_O_2_ to EHL, and 0.01 mass ratio of Fe(OH)_3_ to EHL. The hydroxyl content of HEHL was prepared under the above conditions, which change as shown in Table 3. The alcohol hydroxyl content, phenolic hydroxyl content, and total hydroxyl content of HEHL were 2.94%, 1.05%, and 3.99% higher than those of EHL. Given the satisfactory results, the above experimental conditions were not optimized further and were applied to subsequent experiments.

#### 3.1.4. Chemical Composition and Structure of EHL

**Figure 4:**
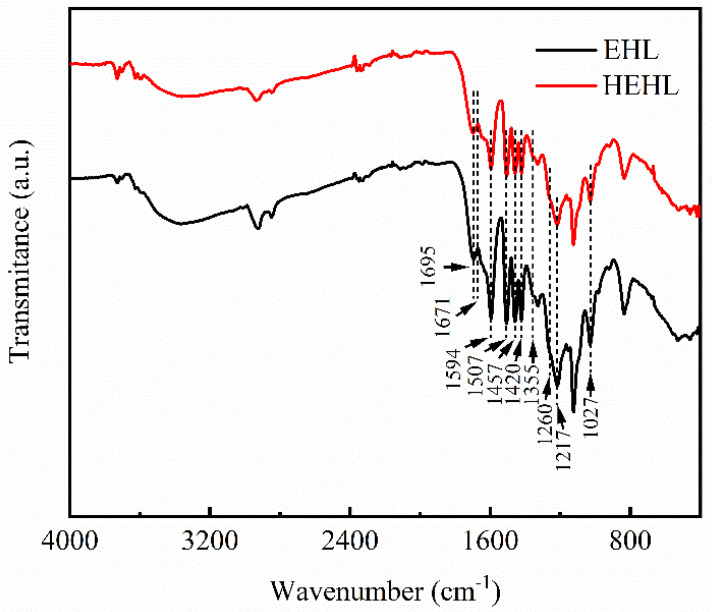
Fourier transform infrared (FTIR) spectra of EHL and HEHL in the 4000–400 cm^−1^ range.

Figure 4 shows the FTIR spectra of the EHL and HEHL spectra, and Table 4 lists the absorption peaks. From Figure 4, it can be seen that the width of the absorption peak of the stretching vibration of the hydroxyl group (‒OH) of HEHL did not vary significantly in the range of 3500–3340 cm^−1^ [25]. Therefore, it needs to be proven by other testing methods. A comparison of the four absorption peaks at 1594, 1507, 1457, and 1420 cm^−1^ [26] shows that the aromatic ring skeleton of HEHL is the same as that of EHL, and only the linkage bonds between the aromatic rings are broken. The increase in hydroxyl content was also verified by analyzing the organic element content (Table 5).

#### 3.1.5. Thermostability Analysis of EHL

**Figure 5:**
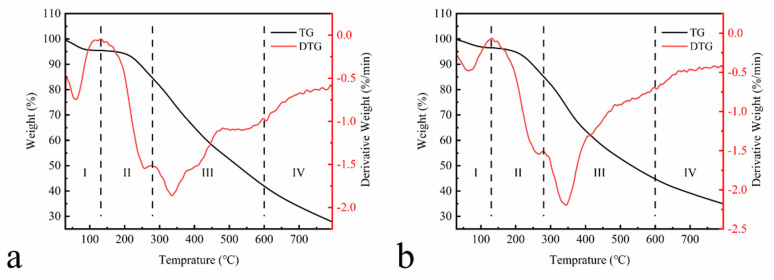
Thermogravimetric analysis of (**a**) EHL and (**b**) HEHL in the temperature range of 30–795 °C with a heating rate of 10 °C/min under nitrogen atmosphere (10 mL/min).

Figure 5 shows the TGA results of EHL and HEHL.

The TGA of lignin samples was performed between 30 and 795 °C under a nitrogen atmosphere. The thermal weight loss process of the samples was divided into four stages. In Stage I (30–130 °C), EHL and HEHL have the same trend. This is attributed to the evaporation of adsorbed water, crystalline water, and organic small molecule components in the lignin. At 60 °C, the maximum dehydration rate of EHL and HEHL reached 0.745 and 0.478%/min. In Stage II (130–280 °C), the weight loss of lignin is related to the bond breakage of the lower dissociation energy in the lignin structure. In stage III (280–600 °C), lignin degrades gradually with the increasing temperature. There is a 42.61% and 40.36% weight loss of EHL and HEHL, respectively, in the third stage from 280 to 600 °C, which is the most significant range of thermal decomposition. The presence of a large number of linkages (e.g., hydroxyl, carbonyl, and other linkages) in lignin results in a wide range of EHL degradation temperatures. At 335.09 °C, the weight loss rate of the EHL reaches the maximum value of 1.86%/min. Moreover, the weight loss rate of HEHL reached a maximum value of 2.16%/min at 333.19 °C, indicating increased intramolecular hydrogen bonding and structural changes. Therefore, HEHL has slightly improved thermal stability compared to EHL. In Stage IV (600–795 °C), the TGA curve stabilizes. The breakage and condensation of aromatic rings mainly occurred in this stage. However, the weight loss is not significant, suggesting that the thermal decomposition process is over. The percentage of residual carbon after thermal degradation of EHL and HEHL was 27.88% and 34.98%, respectively.

### 3.2. Characterization of MFP

#### 3.2.1. Micromorphology of MFP

**Figure 6:**
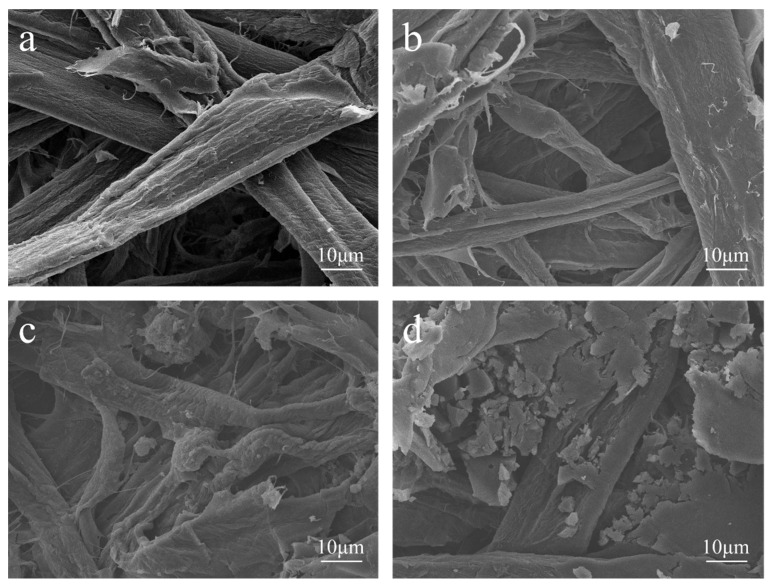

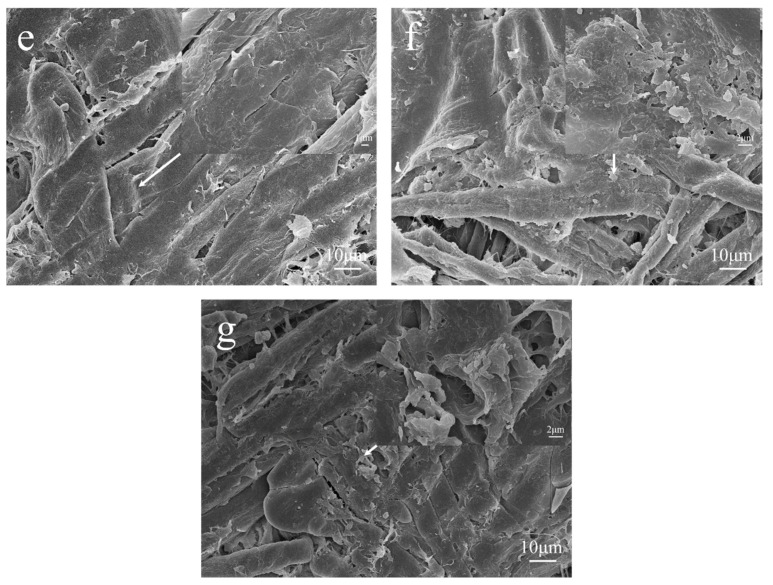
SEM images of the various MFP samples at ×2000 magnification: (**a**) CS; (**b**) CCS; (**c**) ES; (**d**) HES; (**e**) LS; (**f**) ELS; (**g**) HELS.

Figure 6 shows SEM images of the MFP captured to investigate the bonding of lignin during the modification of MFP by EHL composite Lewis acid.

As shown in Figure 6a,b, the CCS fibers are tightly interwoven and have a smooth surface compared with the CS fibers, indicating that the addition of CPAM as a filtering agent did not change the basic morphology of the MFP. It is presumed that the addition of CPAM does not affect the bonding strength between fibers. As shown in Figure 6a,c,d, a layer of lignin was compounded on the surface and between the fibers of ES and HES fibers uniformly than on the CS. The different lignin micromorphology on the fiber surface is attributed to the different hydroxyl content of EHL and HEHL, which also leads to a different enhancement of the mechanical strength of the MFP. As shown in Figure 6a,e, compared to CS, LS fibers modified with Lewis acid during the molding process showed scale-like microstructures due to hemicellulose degradation on the surface, which facilitated the hydrogen bonding between fibers. Comparable to CS, lignin was more uniformly distributed on the fiber surface and between the fibers according to the scale-like microfiber structure of Lewis acid-modified fibers, as shown in Figure 6a,f,g.

#### 3.2.2. Chemical Composition and Structure of MFP

**Figure 7:**
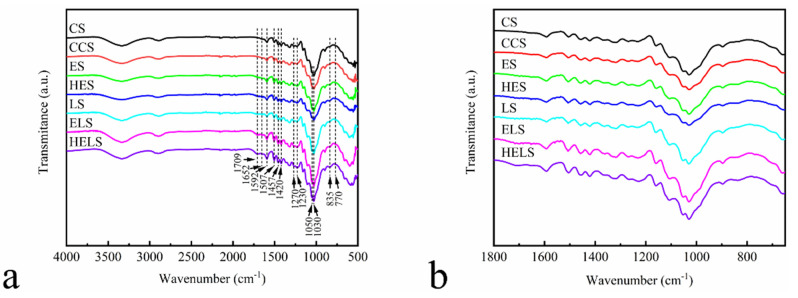
Fourier transform infrared (FTIR) analyses of MFP in the 4000–500 cm^−1^ range: (**a**) Full FTIR spectrogram; (**b**) FTIR spectrogram between 1800 and 650 cm^−1^.

The mechanical performance of an MFP depends on its chemical structure. Therefore, we verified the cross-linking properties of EHL with Lewis acid-modified fibers by FTIR spectroscopy. Figure 7 shows the FTIR spectra of CS, CCS, ES, HES, LS, ELS, and HELS, and Table 6 summarizes the assignments of each spectrum, according to the results reported in [27,28,29]. The absorption peaks of the above samples in the range of 3500–3200 cm^−^^1^ were assigned to the stretching vibration of ‒OH. The broad absorption peaks at 1709 and 1652 cm^−1^ in the MFP are related to the non-conjugated and conjugated C=O stretching vibration, respectively. This was attributed to the degradation of some hemicellulose into furfural by Lewis acid during the molding process and its own condensation. The peaks at 1592, 1507, 1457, and 1420 cm^−1^ belong to the characteristic vibration of the aromatic and furan rings. The absorption peak intensity of CCS was lower than that of CS owing to the addition of CPAM on the fiber surface. The signal of the above absorption peaks is enhanced by the presence of aromatic rings in the structure of the lignin added in ES and HES. The degradation of some hemicelluloses in the Lewis acid-modified fibers to furfural as a furan ring structure led to the enhancement of the above absorption peak signal in LS compared with that of CS. For the ELS and HELS, in a Lewis acid environment, lignin reacts with furfural produced during the molding process to form a phenolic resin-like structure and further enhance the above signal peaks. Peaks at 1270, 1230, 1050, and 1030 cm^−1^ belong to the characteristic vibration of the C‒O stretching vibration peaks. The appearance of the above absorption peaks proves that the lignin added during the molding process introduces a phenolic cross-linking system by reacting with furfural generated by the degradation of hemicellulose. The ELS and HELS spectra have characteristic absorption peaks at 835 and 770 cm^−1^ attributed to the nucleophilic substitution reaction of lignin [30]. This suggests that a phenolic resin-like structure is generated during the molding process.

#### 3.2.3. Thermostability of MFP

**Figure 8:**
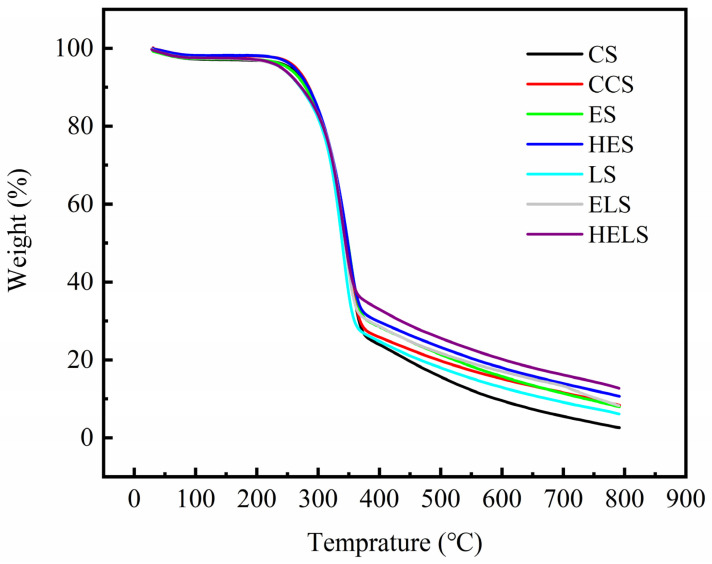
Thermogravimetric analysis of MFP. The temperature was increased from 30 to 795 °C at a heating rate of 10 °C/min under nitrogen atmosphere (30 mL/min).

The TGA curves of CS, CCS, ES, HES, LS, ELS, and HELS are shown in Figure 8. The results showed that the pyrolysis efficiency of all MFP samples was basically the same in the temperature range of 30–795 °C. As shown in Figure 8, the trends of TGA curves for all samples were generally consistent. There is 2–3% weight loss in the first segment from 30 to 115 °C. This is attributed to the evaporation of residual water, physical adsorption water, and crystalline water in the MFP samples. A platform with no weight loss can be obtained in the range of 115 to 200 °C, as the water of the MFP sample has completely evaporated, and no pyrolysis of the MFP sample occurs. As the temperature increases, the MFP samples gradually undergo thermal degradation. There is an approximately 65% weight loss in the second segment from 200 to 380 °C, which is the most significant range of thermal decomposition. Under such heat conditions, glucosyl, and glycosidic bonds, are dramatically cleaved. Small molecules of gases including carbon monoxide and carbon dioxide are released. The TGA curve decreases rapidly, and the weight loss rate increases gradually. Subsequently, the deep thermal degradation of the fibers occurred from 380 to 795 °C, and the pyrolysis of the residual material continued. For lignin, furfural and its condensates are mainly produced in this stage. However, the final residual material content varied from sample to sample. The residual material of HELS was higher than that of CS due to the structure of the furfural-generating resin produced by the degradation of lignin and hemicellulose.

#### 3.2.4. Mechanical Properties of MFP

**Figure 9:**
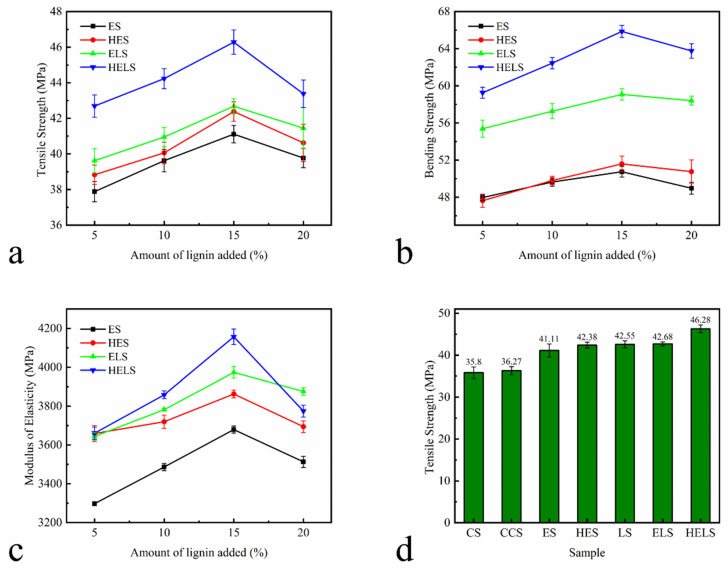

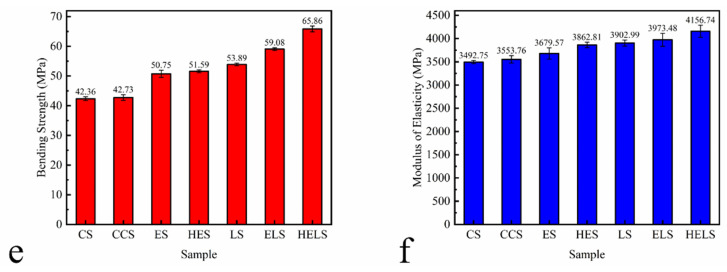
Mechanical Properties of MFP: (**a**) tensile strength, (**b**) bending strength, and (**c**) modulus of elasticity with different amounts of lignin; (**d**) tensile strength, (**e**) bending strength, and (**f**) modulus of elasticity with optimal amount of lignin. All samples were tested using a 1 kN sensor and all data in the Figure 9 are based on three parallel experiments.

The mechanical strength plays a key role in the stability for MFP utilization. The mechanical strengths of MFP with different lignin additions are shown in Figure 9a–c. Form the figure, it can be seen that the optimum amount of lignin was 15%.

The mechanical strength of each sample for the most appropriate lignin addition is shown in Figure 9d–f. The tensile strength, bending strength, and modulus of elasticity of MFP with 15% lignin are listed in Table 7. From Figure 9d–f, and Table 7, it can be seen that the addition of CPAM has basically no effect on the mechanical properties of MFP. With the addition of lignin, the tensile strength of ES and HES increased by 14.90% and 18.42% and their bending strength was increased by 19.81% and 21.79%, respectively. The mechanical strength of the MFP prepared with Lewis acid-modified fibers also increased. The enhancement of the mechanical properties is attributed to the conversion of Lewis degraded hemicellulose to furfural and condensation during the molding process. In summary, a substantial improvement of the mechanical properties of the MFP prepared from lignin composite Lewis acid-modified fibers was observed. The tensile and bending strengths of ELS increased by 19.28% and 39.47%, while those of HELS increased by 29.35% and 55.55%, respectively. The difference in hydroxyl content of lignin is responsible for the difference in strength. The hydroxyl content of HEHL is higher than that of EHL, resulting in a large amount of resin structure generated during the molding process distributed between the fibers. The presence of the resin structure effectively improved the mechanical properties of the MFP.

### 3.3. Mechanical Strength Formation Mechanisms

**Figure 10:**
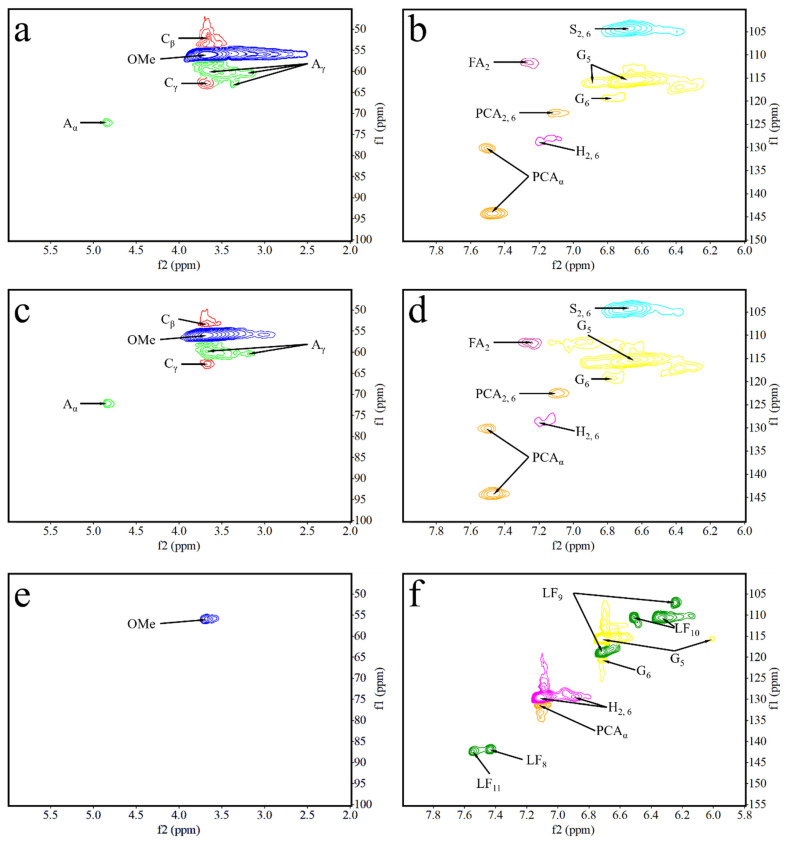
2D-HSQC spectra of EHL, HEHL, model compounds samples: (**a**) Side-chain (δ_C_/δ_H_ 45−100/2.00−6.00) regions of EHL, (**b**) aromatic (δ_C_/δ_H_ 100−150/6.00−8.00) regions of EHL, (**c**) Side-chain (δ_C_/δ_H_ 45−100/2.00−6.00) regions of HEHL, (**d**) aromatic (δ_C_/δ_H_ 100−150/6.00−8.00) regions of HEHL, (**e**) Side-chain (δ_C_/δ_H_ 45−100/2.00−6.00) regions of model compounds, and (**f**) aromatic (δ_C_/δ_H_ 100−150/6.00−8.00) regions of model compounds.

**Figure 11:**
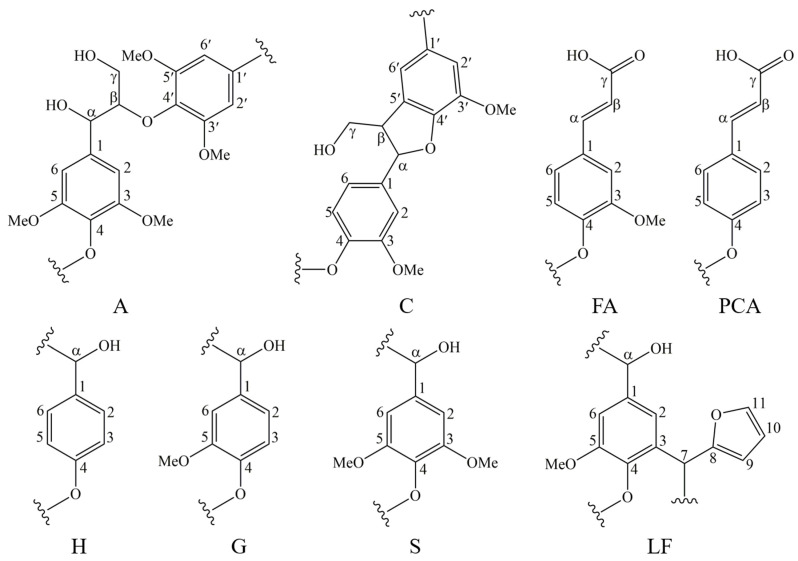
The main structures and linkages in EHL, HEHL, and model compounds: (A) β-O-4′ structures, (C) resinol structures formed by β−β′ coupling, (FA) ferulate units, (PCA) p-coumarate units, (H) p-hydroxyphenyl units, (G) guaiacyl units, (S) syringyl units, and (LF) lignin-furan ring structures.

In order to verify whether the resin structure was generated during the molding process, we compounded polyxylose as a model compound with lignin under the same molding conditions and examined it by 2D-HSQC. The oxygenated aliphatic side chain (δ_C_/δ_H_ 45−100/2.0−6.0) and the aromatic (δ_C_/δ_H_ 100−150/6.0−8.0) regions of lignin samples and the model compounds are shown in Figure 10. 2D- HSQC cross-peaks were assigned by comparison with those reported in the literature [31,32,33,34,35,36,37,38]. The signal assignments are shown in Table 8 and Figure 11 shows the main lignin and model compound structures identified.

The HSQC spectra corresponding to the aliphatic oxygenated regions (δC/δH 45−100/2.0−6.0 ppm) of EHL, HEHL, and model compounds are presented in Figure 10a,c,e, respectively. In Figure 10a,c, the most abundant inter-unit linkages were of the β-O−4′ ether-type (structure A, Figure 11), β-5′ type (structure C, Figure 11), and -OMe of EHL and HEHL. In this region, the cross-peaks (Figure 10) and signal assignments (Table 8) are essentially the consistency, but the strength of signals of β-O−4′ and -OMe of EHL were higher than HEHL. The phenomenon indicates that the -OMe and β-O-4 ether bonds of lignin are broken during the hydroxylation modification process, resulting in a decrease in the molecular weight of lignin. For the model compounds (Figure 10e), within this region only -OMe was present and the signal strength was considered weak compared with EHL and HEHL. This phenomenon indicates that during the hot-pressing process, some chemical bonds of the HEHL are broken or chemically changed to created new chemical bonds.

**Figure 12:**
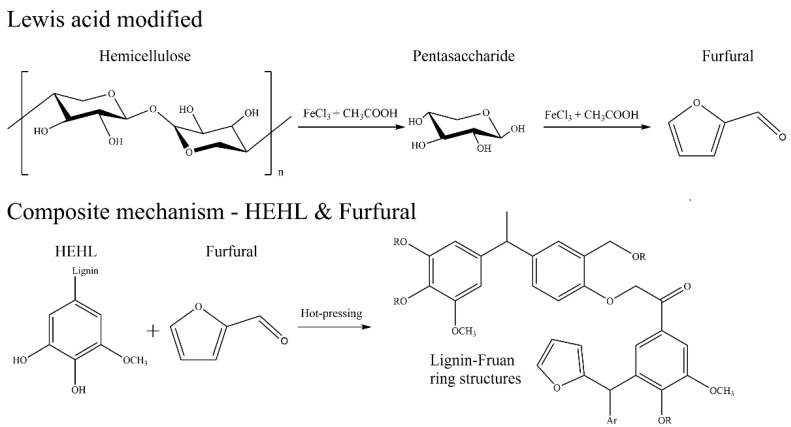
Reaction mechanisms that improve the mechanical properties of MFPs.

The HSQC spectra corresponding to the aromatic regions (δ_C_/δ_H_ 100−150/6.0−8.0 ppm) of EHL, HEHL, and model compounds are presented in Figure 10b,d,f, respectively. In Figure 10b,d, signals from p-hydroxycinnamyl (H), guaiacyl (G), syringyl (S), p-coumarates (PCA), and ferulates (FA) units were observed in the EHL and EHL, respectively. And the signal assignments were shown in Table 8. The main cross-signals of the EHL and HEHL essentially the same in this region. However, in Figure 10f, the characteristic signal of furan ring (LF, Table 8, Figure 11) appears in addition to the above cross-signals. From the above analysis, it is clear that hemicellulose is converted to furfural during hot-pressing under Lewis-acid catalytic conditions. In an acidic environment, the phenolic hydroxyl group on the HEHL condenses with furfural generated in Lewis-acid modification process to generate a phenolic resin structure; the generation of this structure improves the mechanical strength of the MFP. The reaction mechanisms are shown in Figure 12.

## 4. Technical Challenges of MFPs

MFPs are widely used as a cushioning material for medical supplies, daily chemical products, and electronic products owing to the abundance of raw material sources, their low price, and their biodegradability. However, the high cost of mold manufacturing has limited the development of MFPs. Therefore, considering its versatility when designing molds will effectively reduce the cost and expand the scope of MFP applications. Furthermore, some manufacturers use primary pulp as raw materials, which increases the production cost and reduces environmental friendliness. This issue could be solved by using secondary fiber as a raw material to produce MFPs. To promote the use of MFPs in the packaging of large size and heavy weight products and equipment, the raw material ratios, mold design, and compounding of other materials should be optimized.

## 5. Conclusions

Based on the results of this study, the following conclusions can be drawn.

1. Upon modification by hydroxylation, the alcohol hydroxyl content, phenolic hydroxyl content, and total hydroxyl content of lignin increased from 2.24% to 5.18%, from 3.11% to 4.16%, and from 5.35% to 9.34%, respectively. Hydrogen peroxide treatment made the surface of lignin powder became smooth, and the molecular weight decreased significantly.

2. HELS prepared from HEHL compounded with Lewis acid-modified fibers showed a slight decrease in density and a significant increase in mechanical strength compared to CS. Its tensile and bending strengths increased from 35.78 to 46.28 MPa (29.35%) and from 42.36 to 65.86 MPa (55.55%), respectively. Although the thermal stability of HELS was slightly decreased, the increased amount of residual carbon proved that HEHL had been compounded on the surface of Lewis acid-modified fibers and created a resin structure between the fibers, which bonded the fibers more tightly.

3. Milder fiber preparation and modification conditions led to higher mechanical strength of MFP with higher fiber yield and lower production cost, which is beneficial to expand the application of MFPs and lay the foundation for the application of high lignin content fibers.

## Figures and Tables

**Table 1:** Names given to the control group samples.

Sample	Abbreviation
CTMP fibers	CS
CTMP fibers & CPAM	CCS
CTMP fibers, CPAM & EHL	ES
CTMP fibers, CPAM & HEHL	HES
Lewis acid-modified CTMP fiber	LS
Lewis acid-modified CTMP fiber, CPAM & EHL	ELS
Lewis acid-modified CTMP fiber, CPAM & HEHL	HELS

CTMP, chemithermomechanical pulp; CPAM, cationic polyacrylamide; EHL, enzymatic hydrolysis lignin; HEHL, hydroxylated enzymatic hydrolysis lignin.

**Table 2:** Gel permeation analysis for EHL and HEHL.

GPC	EHL	HEHL
M_w_ (g/mol)	16,900	12,600
M_n_ (g/mol)	2900	2800
Distribution index	5.83	4.50

**Table 3:** Hydroxyl content of EHL and HEHL.

Sample	Alcohol Hydroxyl Group (%)	Phenol Hydroxyl Group (%)	Total (%)
EHL	2.24	3.11	5.35
HEHL	5.18	4.16	9.34

**Table 4:** Band Assignments for FTIR of EHL and HEHL.

Wavenumber (cm^−1^)	Assignment
3500–3340	OH groups stretching
2975–2875	C‒H stretching
1695/1671	C=O stretching
1594	C‒C stretching and aromatic vibrations
1507/1457	Aromatic ring vibration
1420	C‒H bending
1355	H‒O bending
1260/1217	Conjugated C‒O
1027	Unconjugated C‒O

**Table 5:** Organic element content of EHL and HEHL.

Sample	N (%)	C (%)	H (%)	S (%)	O (%)
EHL	1.02	62.14	5.58	0.11	29.63
HEHL	1.04	60.67	5.82	0.12	31.27

**Table 6:** Band Assignments for FTIR of MFP.

Wavenumber (cm^−1^)	Assignment
3500–3200	OH groups stretching
2950–2850	C-H stretching
1709	Unconjugated C=O stretching
1652	Conjugated C=O stretching
1592	C‒C stretching and aromatic ring structure stretching
1507/1457	Aromatic and furan ring structure stretching
1420	C‒H bending
1270/1230	Conjugated C‒O stretching
1050/1030	Unconjugated C‒O stretching
835	C‒H out-of-plane bending, para-substituted
770	C‒H out-of-plane bending, ortho-substituted

**Table 7:** Mechanical properties of MFPs.

Sample	Density (g·cm^−3^)	Tensile Strength (MPa)	Bending Strength (MPa)	Modulus of Elasticity (MPa)
CS	0.96	35.78	42.36	3492.75
CCS	0.94	36.27	42.73	3553.76
ES	0.93	41.11	50.75	3679.49
HES	0.96	42.37	51.59	3862.81
LS	0.97	42.55	53.89	3902.99
ELS	0.94	42.68	59.08	3973.48
HELS	0.94	46.28	65.86	4156.74

**Table 8:** Assignment of 13C-1H cross-signals in the HSQC spectra of EHL, HEHL, and model compounds.

Assignment	δC/δH (ppm)		
	EHL	HEHL	Model Compounds
C_β_-H_β_ in phenylcoumarin substructures (C_β_)	53.32/3.50	53.59/3.67	−
C‒H in methoxyls (-OMe)	56.15/3.69	56.08/3.69	55.95/3.68
C_γ_-H_γ_ in β-O-4’ substructures (A_γ_)	60.38/3.59	60.24/3.66	—
—	60.45/3.20	60.56/3.20	—
—	63.12/3.38	—	—
C_γ_-C_γ_ in β-5’ phenylcoumaran substructures (C_γ_)	62.93/3.67	62.89/3.64	—
C_α_-H_α_ in β-O-4’ substructures (A_α_)	72.17/4.84	72.11/4.83	—
C_2, 6_−H_2, 6_ in etherified syringyl units (S)	104.41/6.68	104.17/6.67	—
C_2_-H_2_ in Ferulate (FA)	111.64/7.26	111.60/7.25	—
C_5_-H_5_ in guaicayl units (G)	115.45/6.65	115.32/6.65	115.65/6.01
—	116.10/6.90	—	115.65/6.72
C_6_-H_6_ in guaicayl units (G)	119.51/6.79	119.32/6.76	120.61/6.72
C_2, 6_−H_2, 6_ in p-coumarate (p-CE)	122.58/7.12	122.35/7.09	—
C_2, 6_−H_2, 6_ in p-hydroxyphenyl units (H)	128.80/7.20	128.80/7.2	129.62/7.12
—	—	—	129.52/6.90
C_α_−H_α_ in p-coumarate (p-CE)	130.20/7.50	130.17/7.50	131.38/7.13
—	144.44/7.48	144.25/7.47	—
C_8_ in lignin-furan structure (LF_8_)	—	—	141.93/7.44
C_9_ in lignin-furan structure (LF_9_)	—	—	118.87/6.72
—	—	—	107.15/6.25
C_10_ lignin-furan structure (LF_10_)	—	—	110.56/6.51
—	—	—	110.66/6.33
C_11_ lignin-furan structure (LF_11_)	—	—	142.41/7.55

## Data Availability

The data presented in this study are available on request from the corresponding author.
